# Designing a chimeric subunit vaccine for influenza virus, based on HA2, M2e and CTxB: a bioinformatics study

**DOI:** 10.1186/s12860-020-00334-6

**Published:** 2020-12-04

**Authors:** Davod Jafari, Sara Malih, Mohammad Mahmoudi Gomari, Marzieh Safari, Rasool Jafari, Mohammad Morad Farajollahi

**Affiliations:** 1 Student Research Committee, Faculty of Allied Medicine, Iran University of Medical Sciences, Hemmat Highway, Tehran, Iran; 2 Department of Medical Biotechnology, Faculty of Allied Medicine, Iran University of Medical Sciences, Tehran, Iran; 3 Department of Medical Biotechnology, Faculty of Medical Sciences, Tarbiat Modares University, Tehran, Iran; 4 Department of Microbiology, Faculty of Medicine, Isfahan University of Medical Sciences, Isfahan, Iran; 5 Department of Medical Parasitology and Mycology, Faculty of Medicine, Urmia University of Medical Sciences, Urmia, Iran

**Keywords:** Adjuvant, Bioinformatics, Epitopes, Hemagluttinin, Influenza a virus, Vaccine design

## Abstract

**Background:**

Type A influenza viruses are contagious and even life-threatening if left untreated. So far, no broadly protective vaccine is available due to rapid antigenic changes and emergence of new subtypes of influenza virus. In this study, we exploited bioinformatics tools in order to design a subunit chimeric vaccine from the antigenic and highly conserved regions of HA and M2 proteins of H7N9 subtype of influenza virus. We used mucosal adjuvant candidates, including CTxB, STxB, ASP-1, and LTB to stimulate mucosal immunity and analyzed the combination of HA2, M2e, and the adjuvant. Furthermore, to improve the antigen function and to maintain their three-dimensional structure, 12 different linkers including six rigid linkers and six flexible linkers were used. The 3D structure model was generated using a combination of homology and ab initio modeling methods and the molecular dynamics of the model were analyzed, either.

**Results:**

Analysis of different adjuvants showed that using CtxB as an adjuvant, results in higher overall vaccine stability and higher half-life among four adjuvant candidates. Fusion of antigens and the CTxB in the form of M2e-linker-CTxB-linker-HA2 has the most stability and half life compared to other combination forms. Furthermore, the KPKPKP rigid linker showed the best result for this candidate vaccine among 12 analyzed linkers. The changes in the vaccine 3D structure made by linker insertion found to be negligible, however, although small, the linker insertion between the antigens causes the structure to change slightly. Eventually, using predictive tools such as Ellipro, NetMHCpan I and II, CD4episcore, CTLpred, BepiPred and other epitope analyzing tools, we analyzed the conformational and linear epitopes of the vaccine. The solubility, proteasome cleavage sites, peptidase and potential chemical cutters, codon optimization, post translational modification were also carried out on the final vaccine.

**Conclusions:**

It is concluded that M2e-Linker-CTxB-Linker-HA2 combination of chimeric vaccine retains its 3D structure and antigenicity when KPKPKP used as linker and CTxB used as adjuvant.

**Supplementary Information:**

The online version contains supplementary material available at 10.1186/s12860-020-00334-6.

## Background

Influenza viruses belong to a family of RNA viruses, *Orthomyxoviridae*, that are categorized as types A, B, C, and *Thogotovirus*, which among them, only type A and B are clinically relevant for humans disease [1]. One of the deadliest pandemics in history is the 1918 H1N1 flu virus. The pandemic, which spread worldwide, claimed the lives of nearly 50 million people [2]. Influenza A and B have both HA (hemagglutinin) and NA (neuraminidase) proteins that are expressed on the surface of the virus. So far, based on HA and NA surface antigens, 18 HA subtypes and 11 NA subtypes have been observed for type A influenza viruses that are theoretically expected to generate 198 potential new viral subtypes [3]. The M2 antigen of the influenza A virus is also a surface protein that acts as a tetrameric ion channel pump on the surface of the virus. This activity is carried out after the virus entry into the cell and attaching to endosomes to regulate the pH of the virus capsid and pump the protons via a pH-inducible proton transport mechanism [4]. The HA surface antigen also creates a stalk structure at the virus surface that binds the virus capsid to the host cell surface and allows the virus to enter the host cell by the receptor-binding method [5, 6]. Many studies have shown that both HA and M2 proteins are immunogenic for their host. However, various modifications have been utilized to enhance their immunogenicity and production of neutralizing antibodies against these two proteins [7, 8].

There is always the concern that highly pathogenic subtypes of type A influenza viruss, including H5 and H7, may become more virulent to humans by reassortment with other subtypes and result in increased human-to-human transmissibility. Therefore, we need a quick and inexpensive vaccination system to deal with such threats. Several vaccination strategies have been developed for the influenza virus today, including inactivated and attenuated viruses, viral vector-based vaccines as well as DNA vaccines. Until now, only inactivated and live-attenuated vaccines have been used clinically. However, there are some safety concerns about these vaccines, such as the reassortment of inactivated viruses with other viruses and the possibility of producing a high pathogenic new subtypes in the human body. In addition, the conventional production methods of the influenza vaccine in chicken egg cause allergic reactions in the recipient’s body. Furthermore, the presence of retroviruses in eggs raises the concern that these retroviruses may affect inactivated influenza viruses [9, 10]. In addition to inactivated and attenuated vaccines, many studies have been conducted on the development of third-generation vaccines based on viral vectors. However, the possibility of incorporating these vectors into the genome and causing genetic abnormalities and malignancies actually prevents the expanding of such vaccines [11]. Another type of vaccine that is receiving a great deal of attention is subunit vaccines. Today, these vaccines are produced by biotechnology methods, including recombinant DNA technology [12]. These vaccines are produced in various expression systems, such as the *E. coli* (prokaryotic expression system) or eukaryotic expression systems such as yeast [13], HEK-293 and CHO cell lines [14] as well as plant cells [12]. Due to the antigenic property of HA, NA, and M2e proteins, various studies have been done on the development of a subunit vaccine against influenza virus based on these proteins [15]. In addition to these proteins, NP and M1 have also been used to develop subunit vaccines [16].

Nowadays, bioinformatics tools have come to the aid of biology, biotechnology, and medical researchers to perform various in-silico analysis before conducting any experimental and laboratory research [17]. Obviously, with the analysis, the quality and quantity of empirical experiments will be much improved, and these experiments will be conducted rationally, based on the information obtained from bioinformatics analysis. Various bioinformatics tools can be used in the development of a vaccine [18, 19]. In our previous studies, bioinformatics analyses were performed before in vitro development of the chimeric vaccine candidate for the three anthrax, shigellosis, and cholera diseases [12]. Before the expression of the vaccine in a host by biotechnology techniques, bioinformatics analyses were conducted to optimize the gene construct in terms of its stability and half-life as well as to retain the third structure and epitopes intact [20].

Similarly, the aim of this study was to conduct a bioinformatics analysis of a vaccine candidate containing HA antigen and M2 antigen conserved region with CTxB.

## Results

### Selecting the antigen combination and appropriate adjuvant

The sequence information of antigens and adjuvants are shown in Table S1. The Tm (melting temperature) and Ti (melting temperature index) of antigenic fragments (calculated by http://tm.life.nthu.edu.tw/index.htm) showed in Table S2.

ProtParam server was used to find which combination of these antigens and which adjuvant could result in the most stable structure and longest half-life. As shown in Table 1, the results of the analysis of the various combinations, show that the best adjuvant is CTxB. The lower the instability index (II), the higher the stability of the protein, i.e., if II of a given protein is calculated above 40, the protein is considered to be unstable. When the HA2 antigen is placed in the N-terminal region of fusion proteins, the lowest instability index (II = 32.45) is obtained. The two combinations CMH (C = CTxB, M = M2e and H = HA2) and MCH, both have the lowest II, indicating their stability. In addition they have the highest half-life index (30 h). However, because M2e is a small antigenic region and in many cases is referred to be a conserved epitopic region, we chose the M2e-CTxB-HA2 (MCH) combination (to place M2e at the N-terminal of fusion protein).

**Table 1:** Results of analysis of different combinations of antigens with different adjuvants predicted by ProtParam server

Adjuvant	Fusion protein	II	Aliphatic Index	GRAVY	Half-life (hrs/mammalian)
CTxB	MHC	32.78	76.15	0.459	30
	MCH	32.45	76.15	0.459	30
	HCM	32.78	76.15	0,459	30
	CMH	32.45	76.15	0.459	30
	CHM	32.78	76.15	0.459	30
	HMC	32.78	76.15	0.459	30
ASP-1	MHA	39.17	34.55	0.758	4.4
	MAH	38.74	34.55	0.758	4.4
	AMH	38.74	34.55	0.758	4.4
	AHM	39.17	34.55	0.758	4.4
	HAM	39.17	34.55	0.758	30
	HMA	39.17	34.55	0.758	30
LTB	HML	41.62	35.90	0.847	30
	HLM	42.03	35.90	0.847	30
	LHM	42.03	35.90	0.847	4.4
	LMH	41.51	35.90	0.847	4.4
	Mhl	41.62	35.90	0.847	4.4
	MLH	41.51	35.90	0.847	4.4
STxB	SHM	42.75	34.09	0.783	4.4
	SMH	42.14	34.09	0.783	4.4
	HSM	42.75	34.09	0.783	30
	HMS	42.37	34.09	0.783	30
	MHs	42.37	34.09	0.783	4.4
	MSH	42.14	34.09	0.783	4.4

For other adjuvants, most of them showed unstable combinations (II index above 40). For example, all combinations using LTB and STxB adjuvants showed an II index above 40, indicating that their fusion proteins are unstable. As for the ASP-1 adjuvant also shown, the calculated II indeed is less than 40, but all of their combinations showed an II near 40 (II = 39), which was much higher than that of CTxB adjuvant. These results demonstrate that the CTxB fusion proteins is more stable compared to other adjuvants.

### Selection of linker

Since the natural structures of antigens are crucial for their antigenecity, we investigated the effect of linker application using Protparam server. Table 2 presents the results of the linker analysis using Protparam server. As shown, the stability of the structure in the case of the rigid linkers is generally higher than in the case of the flexible linkers (i.e., they have lower II). Among the rigid linkers, the LR5 (Linker-Rigid number 5) with KPKPKP sequence showed the lowest II, and this linker was selected as the best linker and used in subsequent analyses.

**Table 2:** Analysis of different linkers using ProtParam server

	Linker	Sequence	II of the fusion protein	Aliphatic Index	Gravy
**Flexible linkers**	LF1	(G8) GGGGGGGG	35.08	74.92	−0.455
	LF2	(G4S) GGGGS	32.45	76.15	−0.459
	LF3	(G4S)2 GGGGSGGGGS	33.82	74.12	−0.459
	LF4	GSAGSAAGSGEF	29.26	74.92	−0.425
	LF5	KESGSVSSEQLAQFRSLD	34.65	77.08	−0.487
	LF6	EGKSSGSGSESKST	32.26	72.57	−0.537
**Rigid Linkers**	LR1	EAAAK	31.55	77.80	−0.456
	LR2	A (EAAAK)2AA	30.06	77.68	−0.419
	LR3	(XP)n = APAPAP	35.88	77.38	−0.440
	LR4	(XP)n = EPEPEP	35.63	75.74	.527
	LR5	(XP)n:KPKPKP	28.63	75.74	−0.533
	LR6	(XP)n: GPGP	30.32	76.57	−0.470

### Modeling of the selected chimeric vaccines

In order to find a suitable model for the desired antigenic composition, the first step was Blast. Blast results showed that four templates with 5dlm, 4r8w, 5jw3, 3sdy PDB codes are most similar to our antigenic composition.

The results of the structural quality survey revealed that more than 92% of the amino acid residues are in the favored region in the structure with the linker and more than 91% in the favored region of the without-linker structure.

The quality assessment results by ProSA-web server also demonstrated that for linker-containing structure and linker-free structure, the Z-Score is − 4.85 and − 4.93, respectively.

**Fig. 1:**
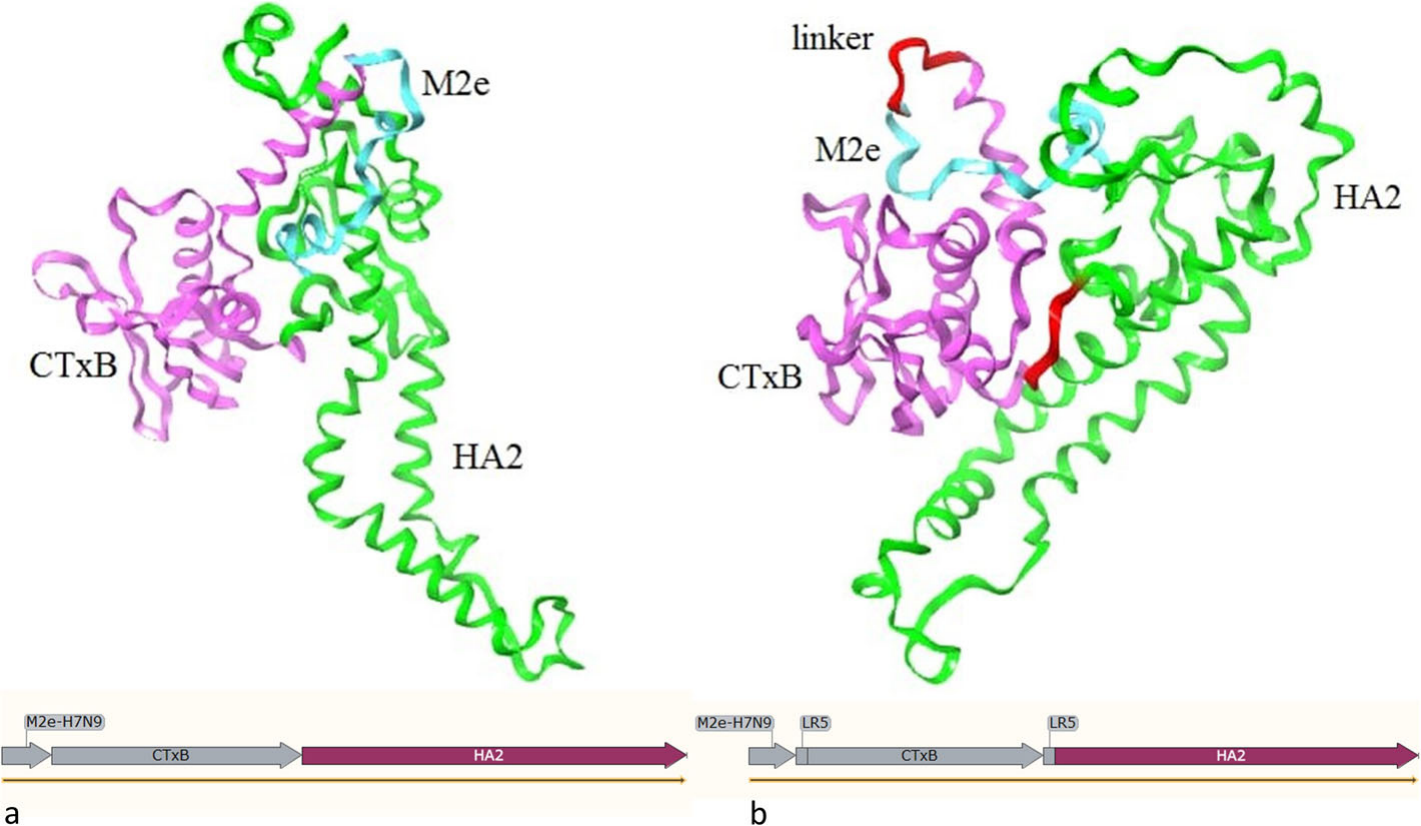
Chimeric vaccine modeling. 3D model of a) linker-free structure (MCH) and b) linker-containing structure (MCH-LR5)

Our final models for the chimeric vaccine were shown in Fig. 1a and b, for MCH and MCH-LR5 structures, respectively.

### Molecular dynamics and free energy calculations

**Fig. 2:**
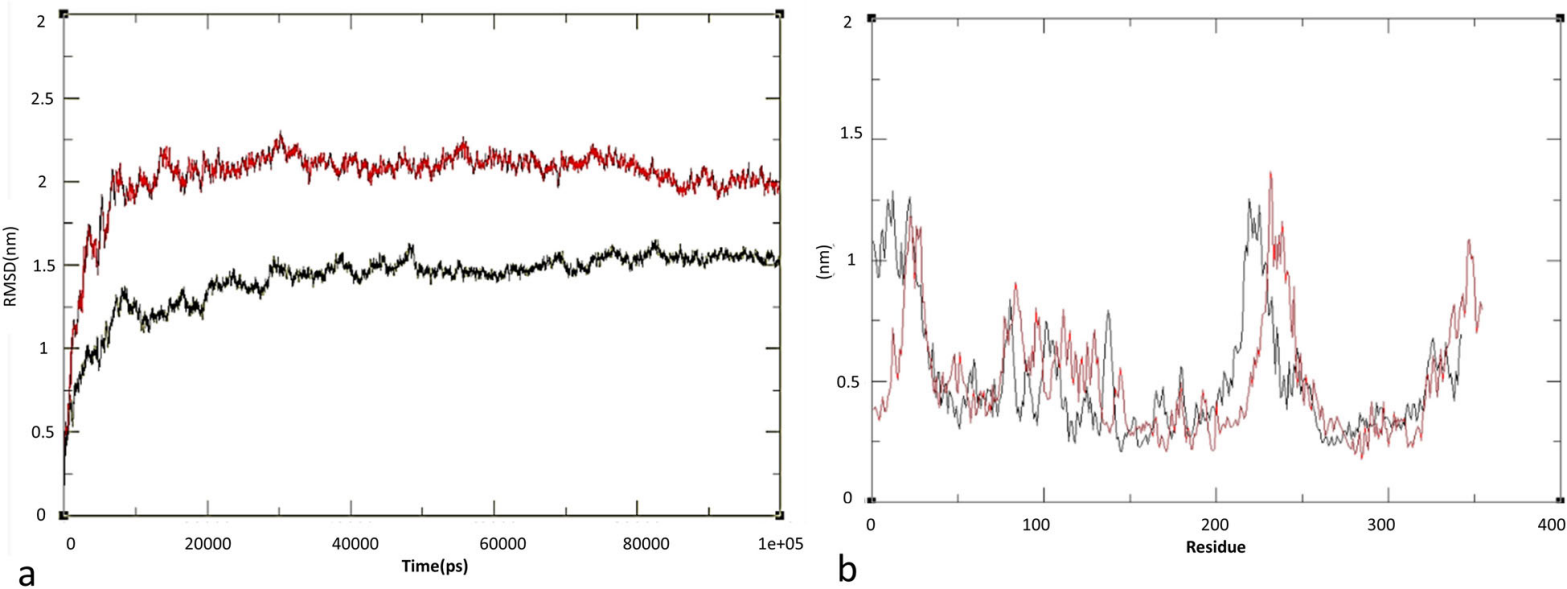
RMSD and RMSF calculation. Calculated **a**) RMSD and **b**) RMSF for modeled structures of chimeric vaccine, MCH (black) and MCH-LR5 (red)

The results of molecular dynamics analysis was shownin Fig. 2. As shown in Fig. 2a for the MHC, the RMSD index was 1.4 Å, and for the MHC-LR5 it was 2.02 Å. Both structures reached equilibrium state in 18 ns. The RMSD difference between these two structures is negligible, but it can be seen from Fig. 2a that although the linker has little effect on the structure, it can increase the flexibility of the structure and modify the relevant antigen behavior. If these changes are significant, the natural structure of the protein undergos changes and can lead to undesirable results. In the case of our antigenic proteins, although their structure unlikely to be completely lost natural state, it potentially can partially alter the structure of antigens and conformational epitopes. In addition, RMSD indexes were calculated separately for M2e, HA2, and CTxB in relation to each other (Table 3). The RMSF index was also calculated to investigate the fluctuations in the structure. This index was calculated for the MCH-LR4 and MCH structures at 0.5 and 0.51 nm, respectively (Fig. 2b). Similar to the RMSD index, there was no significant difference in the RMSF index, but the lower RMSF index of the linker-free structure indicates higher flexibility of construct containing linker.

**Table 3:** RMSD index calculated individually for each antigen in desired structures

Chimeric vaccine	M2e	CTxB	H2A
MCH and MCH-LR5	3.73	8.56	8.29

Gyration Radius analysis showed that the MCH structure had a higher compression rate than the MCH-LR% structure, which is consistent with the results of the RMSF index analysis, which displayed increased flexibility in the structure in which the linker was inserted.

The SASA index was also calculated, which was 215.79 (nm^2^) for the MCH-LR5 structure and 212.2 (nm^2^) for the MCH, respectively. The results obtained during the SASA calculation are in agreement with the outputs of the ROG and show an increase in the water accessibility level of the structure containing the linker and a decrease in its compression. Examination of the secondary structure revealed that there are differences in the secondary structure between the MCH and MCH-LR5, as shown in Table 4.

**Table 4:** Secondary structure information and differences between MCH and MCH-LR5 structures

Linker	Alpha helix	3–10 helix	Pi helix	Extend strand	Beta bridge	turn	Bend	None
MCH-LR5	112	4	20	49	4	49	47	56
MCH	130	10	0	53	3	50	34	61

In order to check the stability of the systems after MD, free energy was estimated by g_mmpbsa, which is an efficient tool. The results are listed in Table 5. Based on the outputs of g-mmpbsa, it can be claimed that the linker can increase the stability of the structure.

**Table 5:** Free energy outputs of constructs using MM-PBSA computations

Energy state	MCH-LR5	MCH
**Potential energy in Vacuum**	− 5598.57	5208.72
Polar solvation energy (kJ mol)	−13,192.5	−12,912.64
Non-polar solvation energy (kJ mol)	515.11	509.628

### Study of epitopes of chimeric vaccine

#### Linear B cell epitope analysis

**Fig. 3:**
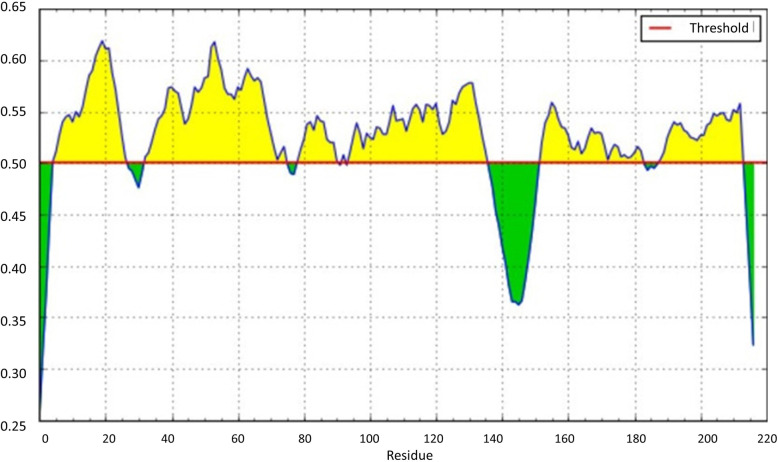
The schematic diagram for epitope regions of M2e-LR5-HA2 antigens ploted by BepiPred server. Epitopic regions are under the threshold curve

Potential linear epitopes in the chimeric vaccine were examined using the BepiPred server. Six potential epitopes less than the threshold (0.5) was found as shown in Fig. 3. The amino acid sequences of these linear epitopes are presented in Table S3. However after confirmation with VaxiJen server with 0.5 threshould (Table S4), Only three linear epitopes seems to be real epitopes with significant antigenicity (Table 6). In addition, predicted epitopes was confirmed with IEDB databases (Table S5).

**Table 6:** Information and sequence of linear epitopes found for B cells predicted by Bepipred and confirmed by VaxiJen

No	Start	End	Sequence	Length
1	5	27	TEVETPTRTGWECNCSGSSDPGL	23
3	79	91	RLIAKTNQQF	13
5	153	183	ENAEEDGTGCFEIFHKCDDNCMASIRNNTYD	31

#### Conformational and linear epitope analysis by Ellipro server

**Fig. 4:**
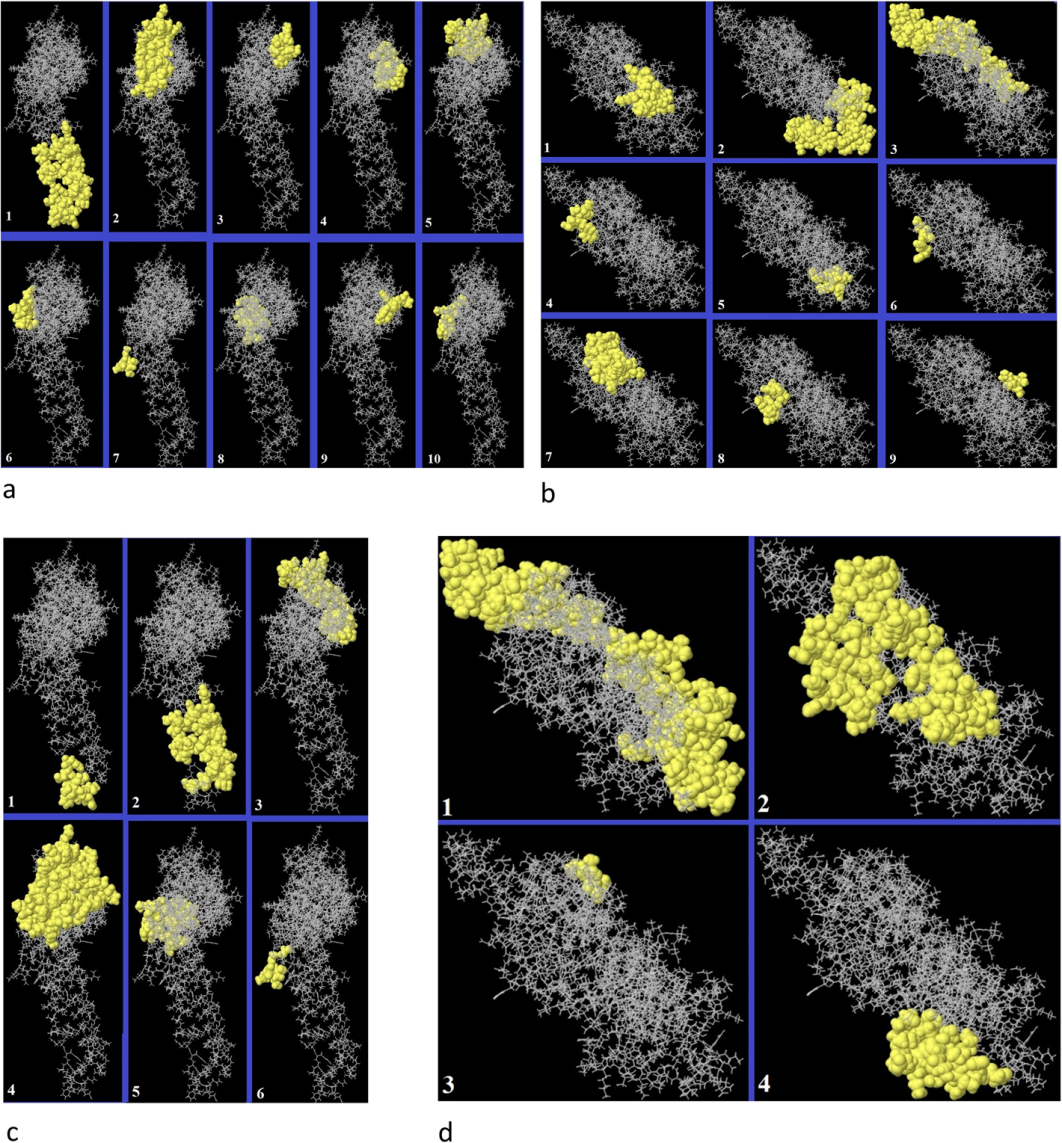
Position of the linear and conformational epitopes of chimeric vaccines. Linear epitopes showed for **a**) MCH (linker free) and **b**) MCH-LR5 (linker-containing) structures of chimeric vaccine. Conformational epitopes showed for **c**) MCH and **d**) MCH-LR chimeric vaccines. The regions showed by yellow color specify the epitopic regions

Both the linear and conformational epitopes for B cells were examined using the Ellipro server. The complete modeled antigenic structures (MCH and MCH-LR5), were analyzed by the server. As listed in Table S9, different linear epitopes were found for the MCH and MCH-LR5 vaccines (Table S6). However, after confirmation by VaxiJen (Table S7) and IEDB (Table S8), only two epitope predicted as real epitope for MCH, and unfortunately no linear epitope was confirmed for MCH-LR5 by this server (Table 7). In the case of the conformational epitopes, 6 and 4 epitopes were found for the MCH and MCH-LR5 structures respectively. All sequence and information of the epitopes are listed in Table S9. The position of the linear and conformational epitopes is depicted in Fig. 4.

**Table 7:** Linear epitopes of MCH and MCH-LR5 predicted by Ellipro and confirmed by vaxijen

Vaccine	No	Start	End	Sequence	Length	Score
MCH	1	202	247	LNRLIAKTNOQFELIDNEFNEVEKOIGNVINVVTROSITEVWSYNAE	46	0.829
	2	68	103	DKIFSYTESLAGKREMAIITFKNGAIFQVEVPGSQH	36	0.741
MCH-LR5	No epitope confirmed by VaxiJen for vaccine containing linker. For details of predicted linear epitopes please see Table S9.

#### CD4 T cells (MHCII) epitope prediction

NetMHCIIpan 0.4 server (http://www.cbs.dtu.dk/services/NetMHCIIpan/) was used for prediction of MHCII binding epitopes. In Table S10, we showed the strong binder epitopes for seven most frequently MHCII alleles (DRB1_0701, DRB1_1501, DRB1_0301, DRB3_0101, DRB3_0202, DRB4_0101, DRB5_0101) for MCH-LR and MCH, respectively. No difference was observed between MHCII epitopes of MCH and MCH-LR5. In addition, these epitopes covered all 7 most frequent alleles of MHCII molecule.

#### Prediction of CTL (MHCI) epitopes

CD8 T cells recognize epitopes presented on MHC class I molecules. The CTLPred and NetMHCIpan 4.01 servers were used to find CTL epitopes. Table 8 and Table S11 show the information of the epitopes of CTL cells predicted by NetMHCIpan and CTLpred, respectively. In Table S12 (for CTLpred) and Table 7 (for NetMHCIpan) MHC Restriction of these epitopes are also shown.

**Table 8:** Predicted epitopes for MHC class I by NetMHCIpan for MCH and MCH-LR

Number	Epitope	HLA Class I allele	Core	Score_EL	%Rank	Bind Level
1	YLTEAKVEKLCV	HLA-A*02:01	YLTEAKVEV	0.1005680	1.878	WB
2	RMKDTLRIAYL	HLA-A*02:01	RMKDTIAYL	0.1528500	1.411	WB
3	LLIAMENQHTI	HLA-A*02:01	LLMENQHTI	0.2700590	0.855	WB
4	KLSSGYKDVIL	HLA-A*02:01	KLSSGDVIL	0.1173450	1.671	WB
5	KLKFGVFFTV	HLA-A*02:01	KLFGVFFTV	0.3885330	0.553	WB
6	TLNDKIFSYT	HLA-A*02:01	TLNDKIFYT	0.1308210	1.551	WB
7	TLRIAYLTEA	HLA-A*02:01	TLIAYLTEA	0.1362920	1.512	WB
8	YLTEAKVEKL	HLA-A*02:01	YLTEAVEKL	0.6496180	0.233	SB
9	GLFGAIAGFI	HLA-A*02:01	GLFGAIAFI	0.1650370	1.323	WB
10	ELIDNEFNEV	HLA-A*02:01	ELIDNEFEV	0.1867090	1.205	WB
11	KLYERVKRQL	HLA-A*02:01	KLYERVKQL	0.3265950	0.689	WB
12	TLNDKIFSY	HLA-A*02:01	TLNDKIFSY	0.1792230	1.243	WB
13	KIFSYTESL	HLA-A*02:01	KIFSYTESL	0.7627700	0.134	SB
14	GLFGAIAGF	HLA-A*02:01	GLFGAIAGF	0.1582900	1.373	WB
15	GLIDGWYGF	HLA-A*02:01	GLIDGWYGF	0.2246050	1.028	WB
16	AIDQITGKL	HLA-A*02:01	AIDQITGKL	0.1138960	1.708	WB
17	LIDNEFNEV	HLA-A*02:01	LIDNEFNEV	0.4518310	0.457	SB
18	IAMENQHTI	HLA-A*02:01	IAMENQHTI	0.2537330	0.911	WB
19	KLSSGYKDV	HLA-A*02:01	KLSSGYKDV	0.0969210	1.929	WB

*WB* Weak binders, *SB* Strong Binders

#### Codon usage optimization

Four expression hosts including *Homo sapiens*, *Mus musculus*, *Saccharomyces cerevisiae* and *Escherichia coli* were used for codon usage optimization. Optimized codones for each host listed in Table S13.

#### Post translational modification prediction

Prediction of post translational modifications (PTMs) carried out for two vaccine candidates. One O-glycosylation site was found for MCH-LR5 at site 152. All possible sites for O-glycosylation were predicted by the neural network (Figure S1). Nevertheless, for MCH-LR5 only one site (152) passes the threshold (0.5), and no O-glycosylation site was found for MCH.

Among five possible N-glycosylation sites, one N-glycosylation was found in the first N of CTxB protein at site 56 and site 50 that passed the threshold (0.5) in MCH-LR5 and MCH, respectively (Figure S2).

Possible phosphorylation PTM in serine, tyrosine and threonine were shown in Figure S3. No significant difference was observed in the pattern of phosphorylation between MCH-LR and MCH.

#### The solubility of the antigenic fusion protein

**Fig. 5:**
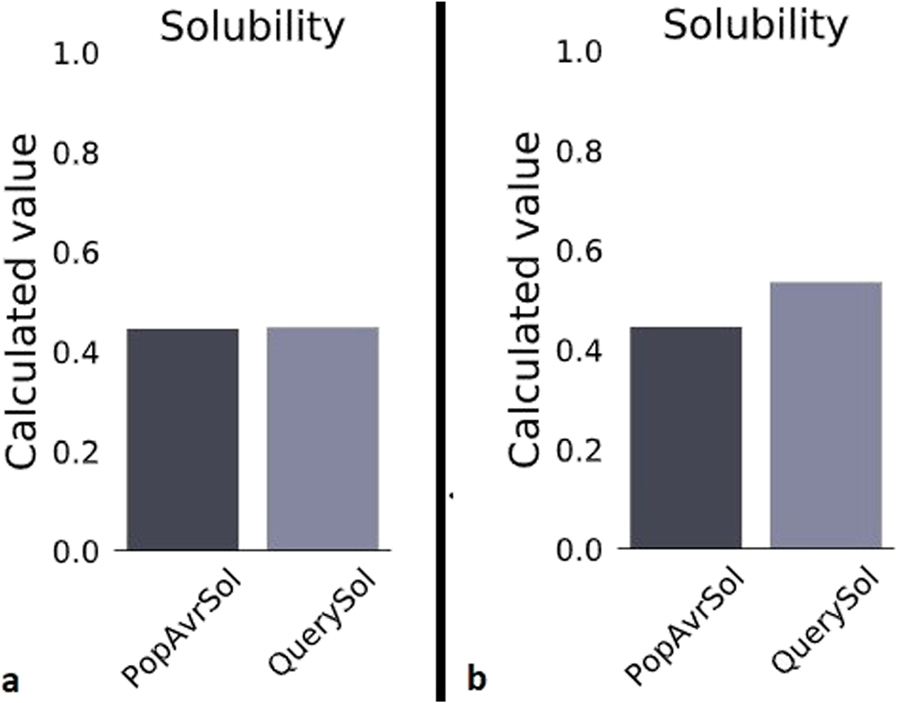
The solubility of antigenic composition (QuerySol column: predicted column), **a**) for the linker-containing mode and **b**) without the linker, compared to the average solubility of the *E. coli* proteins (PopAvrSol column)

The solubility of the complete protein composition, including MCH and MCH-LR5, was calculated using Protein-sol server. The results can be seen in Fig. 5. The solubility of the MCH-LR5 was calculated to be 0.449, which is approximately equal to the average of *E. coli* proteins. PI of this antigenic compound was 6.04.

In the case of MCH, it was found that the solubility was higher than the MCH-LR5 (0.536), and its PI was 5.27. These results indicated that the insertion of a linker (LR5) between antigenic compounds can reduce the fusion protein’s solubility.

### Other in silico physiochemical analysis

#### Immunogenicity of the antigenic compound for a set of seven HLA class II alleles

Using the CD4Episcore server, the CD4 T cell immunogenicity of M2e-LR5-HA2 antigens was predictes for human MHC-II (seven HLA alleles [21]). The combined method and threshold of 50 were used for prediction. The results of this analysis are shown in Table 9.

**Table 9:** Immunogenicity of the antigenic compound (M2e-LR5-HA2) for 7 alles of HLA class II

No	Peptide	Start	End	Imrnunogenicity Score	Peptide core	HLA- DRB1:03:01	HLA- DRB1:07:01	HLA-DRB1:15:01	HLA- ORB3:01:01	HLA- DRB3:02:02	HLA- DRB4:01:01	HLA- DRB5:01:01
1	DSITEVWSYNAELLI	111	125	90.9629	ITEVWSYNA	26.56	13.18	5.61	18.8	43.07	78.93	16.56
2	AMQNRIQJDPVKLSS	191	205	93.6562	RIQIDPVKL	0.02	38.96	24.19	0.97	4.53	5.15	13.28

#### Examination of disulfide bonds

This analysis was done by the DIANNA server. The results of the study of disulfide bonds are shown in Table 10. As shown, the linker entry changes the position of a disulfide bond, one of the contributors to this linkage is cysteine 19 in both structures, but the second contributors in the MCH-LR5 structure is cysteine 306 and in MCH is cysteine 55.

**Table 10:** Results of disulfide bonds analysis of linker-free and linker-containing structures by DIANNA 1.1 Server

	Number	Location	Sequence
**MCH-LR5**	1	17–299	RTGWECNCSGSEDGTGCFEIFH
	2	19–306	GWECNCSGSSDEIFHKCDDNCM
	3	138–310	KVEKLCVWNNKKCDDNCMASIR
**MCH**	1	17–287	RTGWECNCSGSEDGTGCFEIFH
	2	19–55	GWECNCSGSSDNITDLCAEYHN
	3	132–294	KVEKLCVWNNKEIFHKCDDNCM

#### Proteasome cleavage site analysis

The results of the ProteinCutter digestion site analysis are presented in Table S14.

## Discussion

Large epidemics and pandemics have been documented in the history of world by various subtypes of the influenza virus. Even today with the tremendous advances in medicine and biomedical sciences, there is still much concernes about the occurrence of similar epidemics and pandemics [22]. Current vaccines against influenza could not induce long-lasting immunity, so each year, specific vaccine is produced based on the dominant subtypes for the coming year [10]. For this reason, it is necessary to produce subunit vaccines using conserved regions of the virus so that the virus cannot scape from memorized immune responses [23]. Studies showed that the influenza virus HA2 (or stem region) region of HA angigens is more conserved than it’s globular region (HA1). Therefore, HA2 attracted considerable interest for developing a universal vaccine for influenza virus [1]. On the other hand, the M2 antigen has a fragment in its N-terminal region that is highly conserved in almost all influenza virus subtypes [24]. The only disadvantage of M2e is its low immunogenicity, therefore, needs to be used with adjuvants to improve M2e-based vaccines immunogenicity [25]. Studies have used multiple copies M2e and have produced neutralizing antibodies against this region in immunized animals. In some cases, very promising results have been obtained against challenges with different subtypes of virus and observed cross-protection [5, 7].

In this study, we designed a chimeric subunit vaccine for the influenza virus using the conserved regions of HA2 and M2e antigens. As mentioned, because of the low immunogenicity of M2e, application of adjuvants is required. Therefore, in this study, for the first time, four different immunoadjuvant proteins were used from different organisms. These adjuvants have been used in many studies and their adjuvanticity have been confirmed. As noted earlier, in chimeric subunit vaccines or multi-antigenic vaccines (MAVs), several antigens or epitope fragments are put together to provide more potent immunogenicity. Due to the fusion of several fragments together, interference in structures may affect the 3D structure, stability and half-life of the vaccine. Therefore, we assessed all combinations of antigens to find a combination that has most stable and natural state. Recently, in silico study on HA with another adjuvant called Mx fused by EAAK linker was carried out [26]. The difference between our study and the mentioned study is that we examined different types of adjuvants and selected the best one, not just one adjuvant, regardless of its effect on the vaccine’s structural and physiochemical properties. Secondly, we did the same for selecting the linker, and we selected the best linker out of 12 different linkers.

We first combined the two HA2 and M2e antigens in all possible combinations with ASP-1, CTxB, STxB, and LTB adjuvants and evaluated their stability and half-life. Surprisingly, the combination of antigens in all combinations with three out of four adjuvants produced unstable proteins. In this regard, only CTxB could form a stable polypeptide in combination with these two antigens. This result is in line with previous studies that propose CTxB as an ideal adjuvant for the designing of subunit vaccines [12, 20, 27]. CTxB is the B subunit of the cholera toxin and is responsible for transferring the toxic subunit (cholera toxin subunit A or CTxA), through the surface GM1 receptors of mucosal cells into the cell. CTxB individually does not cause any toxicity to the cells and cytotoxicity occurs only when CTxA enters the cell [28]. In this study, we used this adjuvant for two reasons. Firstly, studies showed that the affinity of CTxB to GM1 receptors, acts as delivering moiety to the cell surface, thus increases cellular uptake efficiency and antigen presentation [29]. Secondly, the proper stability of this protein, which can even be administered orally, will increase overall protein stability [30, 31]. It should be noted that the adjuvant itself, is an antigen of *Vibrio cholera*, so it can also induce immunization against *Vibrio cholera* [32, 33]. However, the primary purpose of this study was to design a vaccine against influenza so we ignored epitope prediction for CTxB as well as the linker parts.

Linkers as small amino acid sequences are recently used in the fields of biotechnology, including protein engineering and production of fusion proteins [34]. In this study, we used 12 different types of linkers, including six flexible and six rigid linkers between antigenic compounds with CtxB adjuvant, and examined their stability and half-life. Among these 12 linkers, rigid linkers showed higher stability than flexible linkers, which can be justified by the fact that flexible linkers seems to allow antigenic fragments to fluctuation, they cause a loss of stability [35, 36]. In molecular dynamics analysis, the results were consistent with these results. Therefore, the best and most stable form of the chimeric vaccine was obtained when we used the rigid linker with the KPKPKP sequence and we called this linker LR5. Since this linker is rigid, it makes the whole polypeptide structure more stable. The amino acid proline is also present in other proline linkers (GPGP [20]), and its advantage is that it interferes with the regular secondary structures of proteins in fusion sites. Finally, by selecting this linker and CTxB adjuvant, we used the final construct containing M2e-LR5-CTxB-LR5-HA2 for structural analysis.

Using the combination of homology modeling and ab initio method in protein structure prediction, the 3D structure of the chimeric vaccine was modeled, and molecular dynamics simulations were performed. By comparing the RMSD and RMSF indexes, it was found that insertion of the linker between antigens caused little changes in the structure of the vaccine, which was negligible. Therefore, it is unlikely that this linker will cause significant alterations in the overall structure of the vaccine to disrupt their conformational epitopes.

To investigate epitopes, we mapped linear and conformational epitopes using several tools. Since more than 90% of B cell epitopes are conformational epitopes, these epitopes are critical. We used Bepipredb and Ellipro servers to determine both linear and conformational epitopes. In adddtion, CD4 and CTLs epitopes were also predicted. CD4 epitopes covered all seven most frequent alleles of MHCII molecule. Finally, we analyzed the protein disulfide bond sites, solubility, and immunogenicity using different tools. Codon optimization carried out for four different expression hosts for heterologous protein expression. In addition, PTMs also were predicted for candidate chimeric vaccine. Disulfide bonds and PTMs pattern of this candidate vaccine suggest that the best host choice for protein expression is mammalian cell lines.

Eventually, our results showed that this combination of HA2, M2e, and CtxB could be a suitable candidate chimeric vaccine for further analysis through in vitro and in vivo experiments.

## Conclusions

There are several points to consider when concluding this study. First, although bioinformatics tools have been used in the last two decades to help researchers in the medical and biological sciences, it should be noted that these tools can bring us closer to a goal and they do not provide a definite result. So our research needs to be confirmed in subsequent in vivo experiments. However, this in silico study showed that the two antigens M2e and HA2 can be fused with CTxB by a rigid linker, and this fusion occurs without affecting the stability and vaccine’s 3D structure. Therefore, in the next phase of this study, vaccine’s optimized sequence can be molecularly cloned into an expression vector to express the protein in mammalian cells and investigate its immunogenicity in animals.

## Methods

### Retrieving and evaluating the antigenic sequences

Nucleic acid and protein sequences for the M2 and HA antigens for Influenza A virus (A/chicken/Fujian/SD180/2017(H7N9)) were retrieved from the Nucleotide database at NCBI and analyzed using Snapgene software (https://www.snapgene.com/). Highly antigenic fragments of M2 and HA regions were identified as M2e and HA2 through literature review and their amino acid sequence was used for analysis.

Sequences of several adjuvants were also obtained from the same database and used in subsequent analyses. These adjuvants were CTxB (*Vibrio cholera*) [12], ASP-1 (*Onchocerca volvulus)* [37], LTB (*Escherichia coli)* [38], and STxB (*Shigella dysenteriae)* [39] that recent studies have shown their mucosal immunoadjuvant potential. Protein sequences of antigenic components and adjuvants listed in Table S1.

### Analyzing the different fusion proteins

In order to construct a chimeric vaccine from M2e and HA2 antigens with a suitable adjuvant, different combinations of these proteins were made and analyzed by ProtParam server (https://web.expasy.org/protparam/) to evaluate stability and half-life of resulting fusion proteins.

### Linker selection

In a chimeric vaccine that combines several different protein fragments, linkers act as separators and needed for maintaining the three-dimensional structure of fusion protein. In this research, various linkers were selected from previous studies. Each of these linkers was placed among the antigens selected in the previous step and analyzed by ProtParam server, and the best linker was selected regarding the stability and half-life of the fusion proteins.

### Three dimensional structure modeling

We selected a combination of antigens from the previous analysis with best types of linker and adjuvant as the final construct. In order to investigate the structural variation and the effect of the linker on the antigens’ structure, the chimeric vaccines’ three-dimensional structures was modeled. For this purpose, Modeller software version 9.21 was used to construct the three-dimensional structure of M2e-L-HA2-L-CTxB (L represents linker) and M2e-HA2-CTxB. This software performs homology-based modeling using BLAST. In the first step, to find an appropriate template for our protein structure, BLAST search was performed. As predictable for complex constructs, for some regions of the antigen, structure of the desired template was not found. For this purpose, the Quark server was used to model these areas using the ab initio strategy. Finally, the structures derived from Modeller and Quark software were assembled using PyMod software and the final model was created.

#### Model quality assessment

After the modeling of the structure, the quality of the models was examined. In this regard, the phi (Φ) and psi (ψ) torsion were analyzed by the rampage server. The PROSA-web server was also used to analyze the model quality further.

### Molecular simulation and free energy calculations

Molecular dynamics analysis was performed by Gromacs software 2019.1 in 100 ns to analyze structural stability and structural variations in the model due to the linker addition. To begin this analysis, the topology for both structures (vaccines with and without linker), was first generated by the amber03 force field. Subsequently, the structures were placed inside a Tip3 water box. In the next step, for neutralization of the system, ionization carried out using CL^−^ and NA^+^ ions at a concentration of 0.15 M. Minimization for the system was performed by the Steepest descent minimization method. In the equilibration process, the NVT and NPT steps were taken to couple system to desired temperature and pressure (here 310 K and 1 bar). In the NVT and NPT steps, the V-rescale method and Berendsen method were used, and the time taken to perform these two steps was 200 and 600 ps, respectively. After completing the molecular dynamic step, root-mean-square deviation (RMSD) index was calculated for both structures (with and without linker) to investigate structural changes. The secondary structure was also analyzed to investigate possible changes in this structure with and without linkers. The radius of gyration (ROG) and root mean score of fluctuation (RMSF) indexes were used to analyze protein folding and level of fluctuations to investigate further changes induced by the addition of a linker in the structure of antigens. In the present study using g_mmpbsa module of gromacs, free energy was calculated for our constructs. The free energy calculation procedure by g-mmpbsa comprises three steps. At first potential energy in vacuum is calculated, and then polar, and non-polar solvation energies were computed. For non-polar solvation energy calculation, the solvent-accessible surface area (SASA) model was employed.

### Study of epitopes in the chimeric vaccine

In order to map epitopic regions as well as their antigenic quality in a vaccine, in vitro and in vivo experiments are necessary. However, bioinformatics has provided some tools that help to identify and analyze epitopes and antigenic states of a designed vaccine. In this study, bioinformatics tools were used to recognize humoral and cellular immune system epitopes for the designed chimeric vaccine. As previous studies have shown, many bioinformatics tools can map epitopes on B cells, T cells, and CTLs. Epitopes are classified into two main types of linear and conformational epitopes, we investigated both types of epitopes in this study. Linear epitopes, as the name implies, are epitopes that exist in a continuous sequence and can be easily screened by a series of software and servers using antigen or vaccine sequences. However, the most important type of epitopes, especially in studies requiring the induction of neutralizing antibodies, are conformational epitopes that may be composed of a set of long-distance amino acids. Therefore, for the investigation and mapping of these epitopes, it is necessary to predetermine the exact and high-resolution structure of the 3D antigen or vaccine by the modeling software.

#### Linear epitope prediction for B cells

Bepipred 2.0 server (http://www.cbs.dtu.dk/services/BepiPred/), was used to investigate linear epitopes of B cells. The threshold used for prediction on this server was set to 0.5.

#### Conformational epitopes of B cell

The Ellipro server (http://tools.iedb.org/ellipro/) can predict the conformational epitopes for antibodies using the 3D structure. The PDB file created by the modeling method was given to the server as an input for both with (MCH-LR5) and without linker (MCH) structures. It should be noted that in this server, we had to remove water molecules from the structure, so the water molecules were removed from the model. The software settings were set by default to 0.5 for minimum score and 6 for maximum distance.

#### MHC class I and class II epitope prediction

Epitopes related to CTL cells (MHCI) were examined using the CTLPred server (http://crdd.osdd.net/raghava/ctlpred/) and NetMHCIpan 4.01 server (http://www.cbs.dtu.dk/services/NetMHCpan/). NetMHCIpan server uses ANN for prediction of molecules binding to any HLA class I molecules. In this analysis 8–11 aa was selected for the length of epitopes. In addition class I HLA super-type alleles were used for epitope prediction. The maximum %rank for weak and strong binders set as 2 and 0.5, respectively.

As CD4 T cell recognizes epitopes presented on MHCII molecules, NetMHCIIpan 0.4 server (http://www.cbs.dtu.dk/services/NetMHCIIpan/) was used for prediction of MHCII binding epitopes. This server uses ANN for prediction of any 15 aa length of a protein sequence that binds to any MHC II alleles. For strong binders we set the %rank < 2 and for weak binders < 10. Strong binders for seven most frequently MHCII alleles (DRB1_0701, DRB1_1501, DRB1_0301, DRB3_0101, DRB3_0202, DRB4_0101, DRB5_0101) were predicted.

#### Codon usage optimization for heterologous expression of designed chimeric vaccine candidate

Codon usage for MCH-LR5 as the candidate vaccine was optimized with Jcat server (http://www.jcat.de/). Four expression hosts including *Homo sapiens*, *Mus musculus*, *Saccharomyces cerevisiae* and *Escherichia coli* were used for codon usage optimization.

#### Post translational modification analysis

Prediction of post translational modifications (PTMs) carried out for two vaccine candidates. NetOglyc4.0 server (http://www.cbs.dtu.dk/services/NetOGlyc/) used for prediction of O-glycosylation sites and NetNglyc1.0 server (http://www.cbs.dtu.dk/services/NetNGlyc/) used for prediction of N-glycosylation sites. In addition, for prediction of potential phosphorylation sites in vaccine, we used NetPhos 3.1 server (http://www.cbs.dtu.dk/services/NetPhos/). We performed the phosphorylation for serine, tyrosine and threonine residues.

### Other analysis of the chimeric vaccine candidate

#### Proteasome cleavage sites

Digestion sites for proteinases and chemicals were determined by the PeptideCutter server (https://web.expasy.org/peptide_cutter/) from Expasy. The server identifies and provides all possible cleavage sites for various types of proteinases and peptidases as well as chemicals.

In addition to PeptideCutter, probable cleavage sites for the proteasome were investigated using the NetChop3.1 server (http://www.cbs.dtu.dk/services/NetChop/). The settings used in this server were in the C-terminal sequence analysis method with a threshold of 0.5.

#### Immunogenicity of the chimeric vaccine

The CD4Episcore server (http://tools.iedb.org/CD4episcore/) was used for this purpose. To compare the effect of the presence or absence of the linker on the immunogenicity of the vaccine, we used complete sequences of the MCH-LR5 and MCH vaccines.

#### Solubility of the chimeric vaccine

The Protein-sol server (https://protein-sol.manchester.ac.uk/) was used to check the protein solubility when expressed in an organism. Experimental data show that the solubility of most *E. coli* proteins is an average of 0.45. Therefore, if the protein solubility is higher than 0.45, it is likely to be more soluble than *E. coli* proteins [40]. This server needs to receive a sequence from the user (in FASTA format) to identify the protein solubility with its computational algorithms.

#### Disulfide bonds analysis

We used the DIANNA server (http://clavius.bc.edu/~clotelab/DiANNA/) for checking disulfide bonds. This server can identify the disulfide bonds of a protein in the following four steps: a) Performs a PSI-Blast to find similar patterns in databases, b) Using the PSIPRED server, models the secondary structure of the protein, c) Predicts the oxidized state of cysteines, d) Using a neural network algorithm (NN), determines the disulfide bonds [41].

## Supplementary Information

**Additional file 1: Table S1.** Sequences for the different antigenic and adjuvant fragments used in this study. **Table S2.** The Tm and Ti of antigens and vaccines. **Table S3.** Information and sequence of linear epitopes found for B cells by Bepipred. **Table S4.** Linear B cell epitopes from BepiPred confirmed by VaxiJene. **Table S5.** Confirmation of linear B cell epitopes from BepiPred by IEDB. **Table S6.** Linear epitopes predicted by Ellipro. **Table S7.** Confirmation of linear epitopes from Ellipro by VaxiJen. **Table S.** Linear epitopes from Ellipro confirmed by IEDB for MCH and MCH-LR5. **Table S9.** Amino acid residues involved in the conformational epitopes of linker-containing structure (MCH-Lr5) and linker-free structure (MCH), predicted by ellipro and analyzed by vaxijen. **Table S10.** Epitope prediction for MHC class II by NetMHCIIpan for MCH and MCH-LR. **Table S11.** Epitopes found for CTL cells. **Table S12.** MHC restriction of epitopes found for CTL cells. **Table S13.** Codon optimized sequences of MCH-LR5 vaccine candidate for expression in different hosts. Figure S1. O-glycosylation site prediction. Using NetOGlyC server, the potential O-glycosylation sites predicted for a) MCH-LR and b) MCH. Figure S2. N-glycosylation site prediction. Using NetNGlyC server, the potential N-glycosylation sites predicted for a) MCH-LR and b) MCH. Figure S3. Phosphorylation site prediction. Using NetPhos server, the potential phosphorylation sites predicted for a) MCH-LR and b) MCH. **Table S14.** Proteinases that potentially can cut the vaccine.

## Data Availability

Input data for the analyses are available from the corresponding author on request.

## Abbreviations

CTxB: Cholera Toxin B subunite
STxB: Shiga Toxin B subunite
LTB: Heat-Labile entrotoxine B subunite
ASP-1: Activation association Protein 1
HA1 and HA2: Hemagluttinin region 1 and 2
