# Supplementary material for: Designing a chimeric subunit vaccine for influenza virus, based on HA2, M2e and CTxB: a bioinformatics study
Source: BMC Mol Cell Biol. 2020 Dec 4;21:89. doi: 10.1186/s12860-020-00334-6 (PMC7716444; doi:10.1186/s12860-020-00334-6)
Supplement: Supplementary file 1 — Additional file 1: Table S1. Sequences for the different antigenic and adjuvant fragments used in this study. Table S2. The Tm and Ti of antigens and vaccines. Table S3. Information and sequence of linear epitopes found for B cells by Bepipred. Table S4. Linear B cell epitopes from BepiPred confirmed by VaxiJene. Table S5. Confirmation of linear B cell epitopes from BepiPred by IEDB. Table S6. Linear epitopes predicted by Ellipro. Table S7. Confirmation of linear epitopes from Ellipro by VaxiJen. Table S. Linear epitopes from Ellipro confirmed by IEDB for MCH and MCH-LR5. Table S9. Amino acid residues involved in the conformational epitopes of linker-containing structure (MCH-Lr5) and linker-free structure (MCH), predicted by ellipro and analyzed by vaxijen. Table S10. Epitope prediction for MHC class II by NetMHCIIpan for MCH and MCH-LR. Table S11. Epitopes found for CTL cells. Table S12. MHC restriction of epitopes found for CTL cells. Table S13. Codon optimized sequences of MCH-LR5 vaccine candidate for expression in different hosts. Figure S1. O-glycosylation site prediction. Using NetOGlyC server, the potential O-glycosylation sites predicted for a) MCH-LR and b) MCH. Figure S2. N-glycosylation site prediction. Using NetNGlyC server, the potential N-glycosylation sites predicted for a) MCH-LR and b) MCH. Figure S3. Phosphorylation site prediction. Using NetPhos server, the potential phosphorylation sites predicted for a) MCH-LR and b) MCH. Table S14. Proteinases that potentially can cut the vaccine. [file 12860_2020_334_MOESM1_ESM.docx]

| **Table S1. Sequences for the different antigenic and adjuvant fragments used in this study** | | | | |
| --- | --- | --- | --- | --- |
| Name | Sequence | Size | Organism | Description |
| M2e | MSLLTEVETPTRTGWECNCSGSSDP | 25 | Influenza A virus | Conserved region of M2 protein, universal antibody, and cross-protection |
| HA2 | GLFGAIAGFIENGWEGLIDGWYGFKHQNAQGEGTAADYKSTQSAIDQITGKLNRLIAKTNQQFE  LIDNEFNEVEKQIGNVINWTRDSITEVWSYNAELLIAMENQHTIDLADSEMDKLYERVKRQLREN  AEEDGTGCFEIFHKCDDNCMASIRNNTYDHRKYREEAMQNRIQIDPVKLSSGYKDVILWFSFG | 192 | Influenza A virus | The most conserved domain of HA protein |
| Asp-1 | MILFIIFPAIVVAVTGYNCPGGKLTALERKKIVGQNNKYRSDLINGKLKNRNGTYMPRGKNMLE  LTWDCKLESSAQRWANQCIFGHSPRQQREGVGENVYAYWSSVSVEGLKKTAGTDAGKSWW  SKLPKLYENNPSNNMTWKVAGQGVLHFTQMAWGKTYKIGCGVATQCDGGRTLIVICHYSPGG  NMVGEVIYHRGNPCKVDKDCYTKKCLSKSGLCRK | 220 | Onchocerca volvulus | Adjuvant |
| CTxB | MIKLKFGVFFTVLLSSAYAHGTPQNITDLCAEYHNTQIYTLNDKIFSYTESLAGKREMAIITFKNG  AIFQVEVPGSQHIDSQKKAIERMKDTLRIAYLTEAKVEKLCVWNNKTPHAIAAISMANL | 125 | Vibrio cholera | Adjuvant |
| LtB | MNKVKCYVLFTALLSSLYAHGAPQTITELCSEYRNTQIYTI?DKILSYTESMAGKREMVIITFKSG  ETFQVEVPGSQHIDSQKKAIERMKDTLRITYLTETKIDKLCVWNNKTPISIAAISMEN | 124 | Escherichia coli | Adjuvant |
| STxB | TPDCVTGKVEYTKYNDDDTFTVKVGDKELFTNRWNLQSLLLSAQITGMTVTIKTNACHNGGGFS  EVIFR | 69 | *Shigella* *dysenteriae* | Adjuvant |

**Table S2. The Tm and Ti of antigens and vaccines**

| Components | Tm | Ti |
| --- | --- | --- |
| HA2 | 55-65 | 0.693 |
| M2e | <55 | -2.231 |
| CTxB | <55 | -0.107 |
| MCH-LR5 | 55-65 | 0.171 |
| MCH | 55-65 | 0.307 |

**Table S3. Information and sequence of linear epitopes found for B cells by Bepipred**

| No | Start | End | Sequence | Length |
| --- | --- | --- | --- | --- |
| 1 | 5 | 27 | TEVETPTRTGWECNCSGSSDPGL | 23 |
| 2 | 33 | 75 | GFIENGWEGLIDGWYGFKHQNAQGEGTAADYKSTQSAIDQITG | 43 |
| 3 | 79 | 91 | RLIAKTNQQF | 13 |
| 4 | 95 | 136 | FNEVEKQIGNVINWTRDSITEVWSYNAELLIAMENQHTIDLA | 42 |
| 5 | 153 | 183 | ENAEEDGTGCFEIFHKCDDNCMASIRNNTYD | 31 |
| 7 | 189 | 214 | EEAMQNRIQIDPVKLSSGYKDVILW | 26 |

| **Table S4. Linear B cell epitopes from BepiPred confirmed by VaxiJene.** | | | | |
| --- | --- | --- | --- | --- |
| NO. | Sequence | Model selected | Threshold | Overall Protective Antigen Prediction |
| 1 | TEVETPTRTGWECNCSGSSDPGL | Virus | 0.5 | 0.8061 ( Probable ANTIGEN ) |
| 2 | GFIENGWEGLIDGWYGFKHQNA  QGEGTAADYKSTQSAIDQITG | Virus | 0.5 | 0.4559 ( Probable ANTIGEN ) |
| 3 | RLIAKTNQQF | Virus | 0.5 | 0.6957 ( Probable ANTIGEN ) |
| 4 | FNEVEKQIGNVINWTRDSITEVWSYNAELLIAMENQHTIDLA | Virus | 0.5 | 0.4731 ( Probable ANTIGEN ) |
| 5 | ENAEEDGTGCFEIFHKCDDNCMASIRNNTYD | Virus | 0.5 | 0.5947 ( Probable ANTIGEN ) |
| 6 | EEAMQNRIQIDPVKLSSGYKDVILW | Virus | 0.5 | 0.3558 ( Probable NON-ANTIGEN ) |

Table S5. Confirmation of linear B cell epitopes from BepiPred by IEDB.

| Sequence | Antigen | Organism | Epitope | Assays | References | Blast% |
| --- | --- | --- | --- | --- | --- | --- |
| TEVETPTRTGWECNCSGSSDPGL | Matrix protein 2 | Influenza A virus | 6 | 27 | 9 | 90 |
| GFIENGWEGLIDGWYGFKHQNAQGEGTAADYKSTQSAIDQITG | Hemagglutinin | Influenza A virus | 23 | 45 | 9 | 90 |
| RLIAKTNQQF | Hemagglutinin | Influenza A virus | 4 | 8 | 3 | 90 |
| FNEVEKQIGNVINWTRDSITEVWSYNAELLIAMENQHTIDLA | Hemagglutinin | Influenza A virus | 12 | 27 | 4 | 90 |
| ENAEEDGTGCFEIFHKCDDNCMASIRNNTYD | Hemagglutinin | Influenza A virus | 6 | 13 | 3 | 90 |
| EEAMQNRIQIDPVKLSSGYKDVILW | Hemagglutinin | Influenza A virus | 4 | 10 | 2 | 90 |

Table S6. Linear epitopes predicted by Ellipro

|  | No | Start | End |  | No of residue | Score |
| --- | --- | --- | --- | --- | --- | --- |
| **Without Linker** | 1 | 202 | 247 | LNRLIAKTNOQFELIDNEFNEVEKOIGNVINVVTROSITEVWSYNAE | 46 | 0.829 |
|  | 2 | 68 | 103 | DKIFSYTESLAGKREMAIITFKNGAIFQVEVPGSQH | 36 | 0.741 |
|  | 3 | 133 | 141 | VWNNKTPHA | 9 | 0.737 |
|  | 4 | 8 | 33 | ETPTRTGWECNCSGSSDPMIKLKFGV | 26 | 0.702 |
|  | 5 | 318 | 342 | QNRIQIDPVKLSSGYKDVILWFSFG | 25 | 0.694 |
|  | 6 | 119 | 126 | RIAYLTEA | 8 | 0.613 |
|  | 7 | 166 | 170 | GLIDG | 5 | 0.598 |
|  | 8 | 305 | 315 | NTYDHRKYREE | 11 | 0.599 |
|  | 9 | 58 | 64 | YHNTQIY | 7 | 0.562 |
|  | 10 | 291 | 298 | FHKCDDNC | 8 | 0.567 |
| **With Linker** | 1 | 19 | 39 | CSGSSDPKPKPKPMIKLK | 18 | 0.782 |
|  | 2 | 302 | 351 | IFHKCODNCMASIRNNTYDHRKYREEAMQNRIQIDPVKLSSGYKDVILWF | 50 | 0.774 |
|  | 3 | 198 | 258 | ADYKSTQSAIDQITGKLNRLIAKTNQQFELIDNEFNEVEKQIGNVINWTRDSITEVWSYNA | 61 | 0.737 |
|  | 4 | 140 | 146 | WNNKTPH | 7 | 0.694 |
|  | 5 | 286 | 292 | RQLRENA | 7 | 0.683 |
|  | 6 | 65 | 68 | HNTQ | 4 | 0.638 |
|  | 7 | 77 | 101 | FSYTESLAGKREMAIITFKNGAIFQ | 25 | 0.638 |
|  | 8 | 103 | 110 | EVPGSQHI | 8 | 0.593 |
|  | 9 | 178 | 182 | GLIDG | 5 | 0.534 |

Table S7. Confirmation of linear epitopes from Ellipro by VaxiJen.

|  | No. | Sequence | Model selected | Threshold | Overall Protective Antigen Prediction |
| --- | --- | --- | --- | --- | --- |
| MCH | 1 | LNRLIAKTNOQFELIDNEFNEVEKOIGNVINVVTROSITEVWSYNAE | Virus | 0.4 | 0.2584 ( Probable NON-ANTIGEN ) |
|  | 2 | DKIFSYTESLAGKREMAIITFKNGAIFQVEVPGSQH | Virus | 0.4 | 0.3670 ( Probable NON-ANTIGEN ) |
|  | 5 | QNRIQIDPVKLSSGYKDVILWFSFG | Virus | 0.4 | 0.7391 ( Probable ANTIGEN ) |
|  | 7 | GLIDG | Virus | 0.4 | N.A |
|  | 8 | NTYDHRKYREE | Virus | 0.4 | 0.2960 ( Probable NON-ANTIGEN ) |
|  | 9 | YHNTQIY | Virus | 0.4 | 0.2903 ( Probable NON-ANTIGEN ) |
|  | 10 | FHKCDDNC | Virus | 0.4 | 0.2151 ( Probable NON-ANTIGEN ) |
| MCH-LR5 | 2 | IFHKCODNCMASIRNNTYDHRKYREEAMQNRIQIDPVKLSSGYKDVILWF | Virus | 0.4 | 0.3573 ( Probable NON-ANTIGEN ) |
|  | 3 | ADYKSTQSAIDQITGKLNRLIAKTNQQFELIDNEFNEVEKQIGNVINWTRDSITEVWSYNA | Virus | 0.4 | 0.4385 ( Probable ANTIGEN ) |
|  | 5 | RQLRENA | Virus | 0.4 | -0.3572 ( Probable NON-ANTIGEN ) |
|  | 6 | HNTQ | Virus | 0.4 | N.A |
|  | 8 | EVPGSQHI | Virus | 0.4 | -0.2621 ( Probable NON-ANTIGEN ) |
|  | 9 | GLIDG | Virus | 0.4 | N.A |
| ** Some epitopes not analyzed by Vaxijen due to occurring within CTxB protein or Linker regions. | | | | | |

|  | No. | Sequence | Antigen | Organism | Epitope | Assays | References | Blast% |
| --- | --- | --- | --- | --- | --- | --- | --- | --- |
| MCH | 1 | LNRLIAKTNOQFELIDNEFNEVEKOIGNVINVVTROSITEVWSYNAE | Hemagglutinin | Influenza A virus | 9 | 21 | 4 | 90 |
|  | 2 | ETPTRTGWECNCSGSSDPMIKLKFGV | Matrix protein 2 | Influenza A virus | 1 | 2 | 2 | 90 |
|  | 3 | QNRIQIDPVKLSSGYKDVILWFSFG | Hemagglutinin | Influenza A virus | 4 | 10 | 2 | 90 |
|  | 4 | NTYDHRKYREE | Hemagglutinin | Influenza A virus | 3 | 9 | 2 | 90 |
|  | 5 | FHKCDDNC | Hemagglutinin | Influenza A virus | 2 | 6 | 1 | 80 |
| MCH-LR5 | 1 | IFHKCODNCMASIRNNTYDHRKYREEAMQNRIQIDPVKLSSGYKDVILWF | Hemagglutinin | Influenza A virus | 4 | 10 | 2 | 90 |
|  | 2 | ADYKSTQSAIDQITGKLNRLIAKTNQQFELIDNEFNEVEKQIGNVINWTRDSITEVWSYNA | Hemagglutinin | Influenza A virus | 17 | 40 | 4 | 90 |
|  | 3 | RQLRENA | Hemagglutinin | Influenza A virus | 8 | 14 | 6 | 90 |
| ** Some epitopes not analyzed by Vaxijen due to occurring within CTxB protein or Linker regions. | | | | | | | | |

Table S8 . Linear epitopes from Ellipro confirmed by IEDB for MCH and MCH-LR5

| **Table S9. Amino acid residues involved in the conformational epitopes of linker-containing structure (MCH-Lr5) and linker-free structure (MCH), predicted by ellipro and analyzed by vaxijen.** | | | | | | | |
| --- | --- | --- | --- | --- | --- | --- | --- |
| Ellipro analysis | | | | | Vaxijen analysis | | |
|  | No. | Residues | Number of residues | Score | Model selected | Threshold | Overall Protective Antigen Prediction |
| **MCH** | 1 | F213, E214, L215, I216, D217, N218, E219, F220, N221 | 9 | 0.977 | Virus | 0.4 | 1.0108 ( Probable ANTIGEN ) |
|  | 2 | L202, N203, R204, L205, 1206, A207, K208, T209, N210, Q211, Q212, E222, V223, E224, K225, Q226, I227, G228, N229, V230, I231, N232, W233, T234, R235, D236, S237, I238, T239, E240, V241, W242, S243 , N245, A24 6, E247 | 36 | 0.803 | Virus | 0.4 | 0.0729 ( Probable NON-ANTIGEN ) |
|  | 3 | T9, P10 , T11, R12, G14, W15, E16, C17, N18, C 19, S20, G21, S22, S23, D24, P15 , M26, I27 , K28, L29, K30, F31, G32, V33, I323, D324, P325, V326, K327, L328, S329, S330, G331, Y332, K333, D334, V335, I336, L337, W338, F339, S340, F341, G342 | 46 | 0.723 | Virus | 0.4 | 0.6663 ( Probable ANTIGEN ) |
|  | 4 | Y58, H59, N60, T61, Q62, Y64, D68, K69, I70, F71, S72, Y73, T74, E75, S76, L77, A78,G79, K80, R81, E82, M83, A84, I85, I86, T87, F88, K89, N90, G91, A92, I93, F94, Q95, V96, E97, V98, P99, G100, S101, Q102, H103, K115, R119, I120, A121, Y122, L123, T124, E125, A126, K127, V133, W134, N135, N136, K137, T138, P139, H140, A141 | 61 | 0.698 | Virus | 0.4 | 0.4584 ( Probable ANTIGEN ) |
|  | 5 | Q180, E278, H292, K293, C294, D295, D296, N297, C298, S301, N304, N305, T306, Y307, D308, H309, R310, K311, Y312, R313 | 20 | 0.56 | Virus | 0.4 | 0.6215 ( Probable ANTIGEN ) |
|  | 6 | G163, G166, L167, I168, D169, G170 | 6 | 0.545 | Virus | 0.4 | -1.0095 ( Probable NON-ANTIGEN ) |
| **MCH-LR5** | 1 | G178, L179, I180, D181, G182, W183, Y184, N190, A191, Q192, A198, D199, Y200, K201, S202, T203, Q204, S205, A206, I207, D208, Q209, I210, T211, G212, L214, N215, L217, I218, A219, T221, N222, Q223, Q224, F225, E226, L227, I228, D229, N230, E231, N233, E234, V235, E236, K237, Q238-, I239-, G240, N241, V242, I243, N244, W245, T246, R247, D248, S249, I250, T251, E252, V253, W254, S255, R286 , Q287, L288, E290, N291, A292, I302, F303, H304, K305, C306, D307,D308, N309, C310, M311, A312, S313, I314, R315, N316, N317, T318, Y319, D3207, H321, R322, K323, Y324, R325, E326, E327, A328, M329, Q330, N331, R332 | 101 | 0.75 | Virus | 0.4 | 0.4306 ( Probable ANTIGEN ) |
|  | 2 | N18, C19, S20, G21, S22, S23, D24, P25, K26, P27, K28, P29, K30, P31, M32, I33, K34, L35, A62, H65, N66, T67, Q68, K75, F77, S78, T80, E81, S82, L.83, A84, G85, K86, R87, M89, I91, T93, K95, N96, G97, A98, I99, Q101, E103, V104, P105, G106, S107, Q108, H109, I110, W140, N141, N1427, K143, T144, P145, H146 | 58 | 0.694 | Virus | 0.4 | 0.2745 ( Probable NON-ANTIGEN ) |
|  | 3 | N257, A258, L261 | 3 | 0.619 | Virus | 0.4 | N.A |
|  | 4 | T9, T11, T13, Q334, I335, D336, P337, V338, K339, L340, S341, S342, G343, Y344, K345, D346, V347, I348, L349, W350, F351, S353, F353 | 23 | 0.607 | Virus | 0.4 | 0.7166 ( Probable ANTIGEN ) |

| **Table S10. Epitope prediction for MHC class II by NetMHCIIpan for MCH and MCH-LR.** | | | | | | |
| --- | --- | --- | --- | --- | --- | --- |
| Number | Epitope | HLA allele | Core | Core_REL | Score_EL | %Rank |
| 1 | LCAEYHNTQIYTLND | HLA- DRB1:07:01 | YHNTQIYTL | 1.000 | 0.621742 | 1.05 |
| 2 | CAEYHNTQIYTLNDK | HLA- DRB1:07:01 | YHNTQIYTL | 1.000 | 0.736097 | 0.59 |
| 3 | AEYHNTQIYTLNDKI | HLA- DRB1:07:01 | YHNTQIYTL | 0.993 | 0.717069 | 0.65 |
| 4 | TLNDKIFSYTESLAG | HLA- DRB1:07:01 | FSYTESLAG | 0.993 | 0.539408 | 1.52 |
| 5 | LNDKIFSYTESLAGK | HLA- DRB1:07:01 | FSYTESLAG | 1.000 | 0.871551 | 0.22 |
| 6 | NDKIFSYTESLAGKR | HLA- DRB1:07:01 | FSYTESLAG | 1.000 | 0.922637 | 0.11 |
| 7 | DKIFSYTESLAGKRE | HLA- DRB1:07:01 | FSYTESLAG | 1.000 | 0.939426 | 0.08 |
| 8 | KIFSYTESLAGKREM | HLA- DRB1:07:01 | FSYTESLAG | 1.000 | 0.818153 | 0.35 |
| 9 | GTAADYKSTQSAIDQ | HLA- DRB1:07:01 | YKSTQSAID | 1.000 | 0.779007 | 0.46 |
| 10 | TAADYKSTQSAIDQI | HLA- DRB1:07:01 | YKSTQSAID | 1.000 | 0.830101 | 0.32 |
| 11 | AADYKSTQSAIDQIT | HLA- DRB1:07:01 | YKSTQSAID | 1.000 | 0.848298 | 0.27 |
| 12 | ADYKSTQSAIDQITG | HLA- DRB1:07:01 | YKSTQSAID | 1.000 | 0.644031 | 0.94 |
| 13 | TLNDKIFSYTESLAG | HLA- DRB1:15:01 | IFSYTESLA | 1.000 | 0.748992 | 0.66 |
| 14 | LNDKIFSYTESLAGK | HLA- DRB1:15:01 | IFSYTESLA | 1.000 | 0.906575 | 0.23 |
| 15 | NDKIFSYTESLAGKR | HLA- DRB1:15:01 | IFSYTESLA | 1.000 | 0.939012 | 0.14 |
| 16 | DKIFSYTESLAGKRE | HLA- DRB1:15:01 | IFSYTESLA | 0.980 | 0.826543 | 0.44 |
| 17 | GKREMAIITFKNGAI | HLA- DRB1:15:01 | IITFKNGAI | 1.000 | 0.542773 | 1.47 |
| 18 | KREMAIITFKNGAIF | HLA- DRB1:15:01 | IITFKNGAI | 1.000 | 0.921380 | 0.18 |
| 19 | REMAIITFKNGAIFQ | HLA- DRB1:15:01 | IITFKNGAI | 1.000 | 0.969263 | 0.05 |
| 20 | EMAIITFKNGAIFQV | HLA- DRB1:15:01 | IITFKNGAI | 1.000 | 0.971283 | 0.05 |
| 21 | MAIITFKNGAIFQVE | HLA- DRB1:15:01 | IITFKNGAI | 1.000 | 0.889800 | 0.28 |
| 22 | AIITFKNGAIFQVEV | HLA- DRB1:15:01 | IITFKNGAI | 1.000 | 0.507778 | 1.64 |
| 23 | DSITEVWSYNAELLI | HLA- DRB1:15:01 | VWSYNAELL | 0.933 | 0.525762 | 1.55 |
| 24 | SITEVWSYNAELLIA | HLA- DRB1:15:01 | VWSYNAELL | 1.000 | 0.706867 | 0.78 |
| 25 | ITEVWSYNAELLIAM | HLA- DRB1:15:01 | VWSYNAELL | 1.000 | 0.670168 | 0.92 |
| 26 | TEVWSYNAELLIAME | HLA- DRB1:15:01 | VWSYNAELL | 0.993 | 0.504867 | 1.66 |
| 27 | EAMQNRIQIDPVKLS | HLA- DRB1:03:01 | IQIDPVKLS | 1.000 | 0.710637 | 0.94 |
| 28 | AMQNRIQIDPVKLSS | HLA- DRB1:03:01 | IQIDPVKLS | 1.000 | 0.923541 | 0.18 |
| 29 | MQNRIQIDPVKLSSG | HLA- DRB1:03:01 | IQIDPVKLS | 1.000 | 0.948860 | 0.09 |
| 30 | QNRIQIDPVKLSSGY | HLA- DRB1:03:01 | IQIDPVKLS | 1.000 | 0.948517 | 0.10 |
| 31 | NRIQIDPVKLSSGYK | HLA- DRB1:03:01 | IQIDPVKLS | 1.000 | 0.882755 | 0.31 |
| 32 | RIQIDPVKLSSGYKD | HLA- DRB1:03:01 | IQIDPVKLS | 1.000 | 0.556900 | 1.70 |
| 33 | GNVINWTRDSITEVW | HLA- DRB3:01:01 | WTRDSITEV | 1.000 | 0.383505 | 1.41 |
| 34 | NVINWTRDSITEVWS | HLA- DRB3:01:01 | WTRDSITEV | 1.000 | 0.498406 | 0.92 |
| 35 | VINWTRDSITEVWSY | HLA- DRB3:01:01 | WTRDSITEV | 1.000 | 0.505970 | 0.89 |
| 36 | INWTRDSITEVWSYN | HLA- DRB3:01:01 | WTRDSITEV | 1.000 | 0.297990 | 1.93 |
| 37 | EAMQNRIQIDPVKLS | HLA- DRB3:01:01 | IQIDPVKLS | 1.000 | 0.355640 | 1.57 |
| 38 | AMQNRIQIDPVKLSS | HLA- DRB3:01:01 | IQIDPVKLS | 1.000 | 0.641627 | 0.53 |
| 39 | MQNRIQIDPVKLSSG | HLA- DRB3:01:01 | IQIDPVKLS | 1.000 | 0.719288 | 0.35 |
| 40 | QNRIQIDPVKLSSGY | HLA- DRB3:01:01 | IQIDPVKLS | 1.000 | 0.725085 | 0.34 |
| 41 | NRIQIDPVKLSSGYK | HLA- DRB3:01:01 | IQIDPVKLS | 1.000 | 0.502302 | 0.90 |
| 42 | TRTGWECNCSGSSDP | HLA- DRB3:02:02 | WECNCSGSS | 1.000 | 0.257185 | 1.74 |
| 43 | RTGWECNCSGSSDPK | HLA- DRB3:02:02 | WECNCSGSS | 1.000 | 0.404964 | 0.92 |
| 44 | NTQIYTLNDKIFSYT | HLA- DRB3:02:02 | YTLNDKIFS | 0.987 | 0.227117 | 2.00 |
| 45 | TQIYTLNDKIFSYTE | HLA- DRB3:02:02 | YTLNDKIFS | 0.993 | 0.290838 | 1.49 |
| 46 | NDKIFSYTESLAGKR | HLA- DRB3:02:02 | FSYTESLAG | 0.927 | 0.290919 | 1.49 |
| 47 | DKIFSYTESLAGKRE | HLA- DRB3:02:02 | FSYTESLAG | 0.960 | 0.361420 | 1.10 |
| 48 | EKLCVWNNKTPHAIA | HLA- DRB3:02:02 | VWNNKTPHA | 0.967 | 0.350790 | 1.16 |
| 49 | KLCVWNNKTPHAIAA | HLA- DRB3:02:02 | VWNNKTPHA | 1.000 | 0.431260 | 0.82 |
| 50 | AADYKSTQSAIDQIT | HLA- DRB3:02:02 | YKSTQSAID | 0.993 | 0.229781 | 1.98 |
| 51 | TEVWSYNAELLIAME | HLA- DRB3:02:02 | WSYNAELLI | 0.993 | 0.303869 | 1.41 |
| 52 | AMQNRIQIDPVKLSS | HLA- DRB3:02:02 | IQIDPVKLS | 1.000 | 0.228362 | 1.99 |
| 53 | MQNRIQIDPVKLSSG | HLA- DRB3:02:02 | IQIDPVKLS | 1.000 | 0.355384 | 1.13 |
| 54 | QNRIQIDPVKLSSGY | HLA- DRB3:02:02 | IQIDPVKLS | 1.000 | 0.394507 | 0.97 |
| 55 | KNGAIFQVEVPGSQH | HLA- DRB4:01:01 | IFQVEVPGS | 0.787 | 0.276803 | 1.30 |
| 56 | NGAIFQVEVPGSQHI | HLA- DRB4:01:01 | IFQVEVPGS | 0.840 | 0.254932 | 1.49 |
| 57 | MKDTLRIAYLTEAKV | HLA- DRB4:01:01 | LRIAYLTEA | 0.973 | 0.225621 | 1.84 |
| 58 | KDTLRIAYLTEAKVE | HLA- DRB4:01:01 | LRIAYLTEA | 0.920 | 0.302623 | 1.10 |
| 59 | AMQNRIQIDPVKLSS | HLA- DRB4:01:01 | IQIDPVKLS | 0.933 | 0.389920 | 0.67 |
| 60 | MQNRIQIDPVKLSSG | HLA- DRB4:01:01 | IQIDPVKLS | 0.993 | 0.552456 | 0.28 |
| 61 | QNRIQIDPVKLSSGY | HLA- DRB4:01:01 | IQIDPVKLS | 0.953 | 0.593319 | 0.22 |
| 62 | NRIQIDPVKLSSGYK | HLA- DRB4:01:01 | IQIDPVKLS | 0.820 | 0.393824 | 0.66 |
| 63 | RIQIDPVKLSSGYKD | HLA- DRB4:01:01 | IQIDPVKLS | 0.727 | 0.231536 | 1.76 |
| 64 | NDKIFSYTESLAGKR | HLA- DRB5:01:01 | FSYTESLAG | 0.800 | 0.552694 | 1.51 |
| 65 | DKIFSYTESLAGKRE | HLA- DRB5:01:01 | FSYTESLAG | 0.653 | 0.717482 | 0.66 |
| 66 | KIFSYTESLAGKREM | HLA- DRB5:01:01 | YTESLAGKR | 0.700 | 0.621892 | 1.08 |
| 67 | IFSYTESLAGKREMA | HLA- DRB5:01:01 | YTESLAGKR | 0.987 | 0.643867 | 0.97 |
| 68 | QKKAIERMKDTLRIA | HLA- DRB5:01:01 | IERMKDTLR | 0.987 | 0.581626 | 1.32 |
| 69 | KKAIERMKDTLRIAY | HLA- DRB5:01:01 | IERMKDTLR | 0.980 | 0.602681 | 1.18 |

**Table S11. Epitopes found for CTL cells**

| Peptide Rank | Start | Sequence | Score |
| --- | --- | --- | --- |
| 1 | 127 | MENQHTIDL | 0.98/1.1413788 |
| 2 | 138 | SEMDKLYER | 0.82/1.0867449 |
| 3 | 62 | DYKSTQSAI | 0.93/0.55619334 |

Table S12. MHC restriction of epitopes found for CTL cells

| 1 | 2 | 3 | MHC Restriction of CTL epitopes |
| --- | --- | --- | --- |
| MENQHTIDL | SEMDKLYER | DYKSTQSAI | HLA-Cw:04:01 |
| MENQHTIDL | SEMDKLYER | DYKSTQSAI | HLA-G |

| **Table S13. Codon optimized sequences of MCH-LR5 vaccine candidate for expression in different hosts.** | | | | |
| --- | --- | --- | --- | --- |
| **Host organism** | **Server** | **CAI** | **GC%** | **Sequence** |
| Homo Sapiens | Jcat | 0.9551 | 62.61 | ATGAGCCTGCTGACCGAGGTGGAGACCCCCACCCGCACCGGCTGGGAGTGCAACTGCAGCGGCAGCAGCGACCCCAAGCCCAAGCCCAAGCCCATGATCA AGCTGAAGTTCGGCGTGTTCTTCACCGTGCTGCTGAGCAGCGCCTACGCCCACGGCACCCCCCAGAACATCACCGACCTGTGCGCCGAGTACCACAACAC CCAGATCTACACCCTGAACGACAAGATCTTCAGCTACACCGAGAGCCTGGCCGGCAAGCGCGAGATGGCCATCATCACCTTCAAGAACGGCGCCATCTTC CAGGTGGAGGTGCCCGGCAGCCAGCACATCGACAGCCAGAAGAAGGCCACGAGCGCATGAAGGACACCCTGCGCATCGCCTACCTGACCGAGGCCAAGG TGGAGAAGCTGTGCGTGTGGAACAACAAGACCCCCCACGCCATCGCCGCCATCAGCATGGCCAACCTGAAGCCCAAGCCCAAGCCCGGCCTGTTCGGCGC CATCGCCGGCTTCATCGAGAACGGCTGGGAGGGCCTGATCGACGGCTGGTACGGCTTCAAGCACCAGAACGCCCAGGGCGAGGGCACCGCCGCCGACTAC AAGAGCACCCAGAGCGCCATCGACCAGATCACCGGCAAGCTGAACCGCCTGATCGCCAAGACCAACCAGCAGTTCGAGCTGATCGACAACGAGTTCAACG AGGTGGAGAAGCAGATCGGCAACGTGATCAACTGGACCCGCGACAGCATCACCGAGGTGTGGAGCTACAACGCCGAGCTGCTGATCGCCATGGAGAACCA GCACACCATCGACCTGGCCGACAGCGAGATGGACAAGCTGTACGAGCGCGTGAAGCGCCAGCTGCGCGAGAACGCCGAGGAGGACGGCACCGGCTGCTTC GAGATCTTCCACAAGTGCGACGACAACTGCATGGCCAGCATCCGCAACAACACCTACGACCACCGCAAGTACCGCGAGGAGGCCATGCAGAACCGCATCC  AGATCGACCCCGTGAAGCTGAGCAGCGGCTACAAGGACGTGATCCTGTGGTTCAGCTTCGGC |
| Mus musculus | Jcat | 0.7198 | 61.39 | ATGAGCCTGCTGACCGAGGTGGAGACCCCCACCAGGACCGGCTGGGAGTGCAACTGCAGCGGCAGCAGCGACCCCAAGCCCAAGCCCAAGCCCATGATCA AGCTGAAGTTCGGCGTGTTCTTCACCGTGCTGCTGAGCAGCGCCTACGCCCACGGCACCCCCCAGAACATCACCGACCTGTGCGCCGAGTACCACAACAC CCAGATCTACACCCTGAACGACAAGATCTTCAGCTACACCGAGAGCCTGGCCGGCAAGAGGGAGATGGCCATCATCACCTTCAAGAACGGCGCCATCTTC CAGGTGGAGGTGCCCGGCAGCCAGCACATCGACAGCCAGAAGAAGGCCATCGAGAGGATGAAGGACACCCTGAGGATCGCCTACCTGACCGAGGCCAAGG TGGAGAAGCTGTGCGTGTGGAACAACAAGACCCCCCACGCCATCGCCGCCATCAGCATGGCCAACCTGAAGCCCAAGCCCAAGCCCGGCCTGTTCGGCGC CATCGCCGGCTTCATCGAGAACGGCTGGGAGGGCCTGATCGACGGCTGGTACGGCTTCAAGCACCAGAACGCCCAGGGCGAGGGCACCGCCGCCGACTAC AAGAGCACCCAGAGCGCCATCGACCAGATCACCGGCAAGCTGAACAGGCTGATCGCCAAGACCAACCAGCAGTTCGAGCTGATCGACAACGAGTTCAACG AGGTGGAGAAGCAGATCGGCAACGTGATCAACTGGACCAGGGACAGCATCACCGAGGTGTGGAGCTACAACGCCGAGCTGCTGATCGCCATGGAGAACCA GCACACCATCGACCTGGCCGACAGCGAGATGGACAAGCTGTACGAGAGGGTGAAGAGGCAGCTGAGGGAGAACGCCGAGGAGGACGGCACCGGCTGCTTC GAGATCTTCCACAAGTGCGACGACAACTGCATGGCCAGCATCAGGAACAACACCTACGACCACAGGAAGTACAGGGAGGAGGCCATGCAGAACAGGATCC AGATCGACCCCGTGAAGCTGAGCAGCGGCTACAAGGACGTGATCCTGTGGTTCAGCTTCGGC |
| Saccharomyces cerevisiae | Jcat | 0.8873 | 42.37 | ATGTCTTTGTTGACTGAAGTTGAAACTCCAACTAGAACTGGTTGGGAATGTAACTGTTCTGGTTCTTCTGACCCAAAGCCAAAGCCAAAGCCAATGATCA AGTTGAAGTTCGGTGTTTTCTTCACTGTTTTGTTGTCTTCTGCTTACGCTCACGGTACTCCACAAAACATCACTGACTTGTGTGCTGAATACCACAACAC TCAAATCTACACTTTGAACGACAAGATCTTCTCTTACACTGAATCTTTGGCTGGTAAGAGAGAAATGGCTATCATCACTTTCAAGAACGGTGCTATCTTC CAAGTTGAAGTTCCAGGTTCTCAACACATCGACTCTCAAAAGAAGGCTATCGAAAGAATGAAGGACACTTTGAGAATCGCTTACTTGACTGAAGCTAAGG TTGAAAAGTTGTGTGTTTGGAACAACAAGACTCCACACGCTATCGCTGCTATCTCTATGGCTAACTTGAAGCCAAAGCCAAAGCCAGGTTTGTTCGGTGC GATAGCTGGCTTCATCGAAAACGGCTGGGAAGGTTTGATCGACGGTTGGTACGGTTTCAAGCACCAAAACGCTCAAGGTGAAGGTACTGCTGCTGACTAC AAGTCTACTCAATCTGCTATCGACCAAATCACTGGTAAGTTGAACAGATTGATCGCTAAGACTAACCAACAATTCGAATTGATCGACAACGAATTCAACG AAGTTGAAAAGCAAATCGGTAACGTTATCAACTGGACTAGAGACTCTATCACTGAAGTTTGGTCTTACAACGCTGAATTGTTGATCGCTATGGAAAACCA ACACACTATCGACTTGGCTGACTCTGAAATGGACAAGTTGTACGAAAGAGTTAAGAGACAATTGAGAGAAAACGCTGAAGAAGACGGTACTGGTTGTTTC GAAATCTTCCACAAGTGTGACGACAACTGTATGGCTTCTATCAGAAACAA AAATCGACCCAGTTAAGTTGTCTTCTGGTTACAAGGACGTTATCTTGTGG TTCTCTTTCGGT |
| Escherichia coli (K12) | Jcat | 1.0 | 48.49 | ATGTCTCTGCTGACCGAAGTTGAAACCCCGACCCGTACCGGTTGGGAATGCAACTGCTCTGGTTCTTCTGACCCGAAACCGAAACCGAAACCGATGATCA AACTGAAATTCGGTGTTTTCTTCACCGTTCTGCTGTCTTCTGCTTACGCTCACGGTACCCCGCAGAACATCACCGACCTGTGCGCTGAATACCACAACAC CCAGATCTACACCCTGAACGACAAAATCTTCTCTTACACCGAATCTCTGGCTGGTAAACGTGAAATGGCTATCATCACCTTCAAAAACGGTGCTATCTTC CAGGTTGAAGTTCCGGGTTCTCAGCACATCGACTCTCAGAAAAAAGCTATCGAACGTATGAAAGACACCCTGCGTATCGCTTACCTGACCGAAGCTAAAG TTGAAAAACTGTGCGTTTGGAACAACAAAACCCCGCACGCTATCGCTGCTATCTCTATGGCTAACCTGAAACCGAAACCGAAACCGGGTCTGTTCGGTGC TATCGCTGGTTTCATCGAAAACGGTTGGGAAGGTCTGATCGACGGTTGGTACGGTTTCAAACACCAGAACGCTCAGGGTGAAGGTACCGCTGCTGACTAC AAATCTACCCAGTCTGCTATCGACCAGATCACCGGTAAACTGAACCGTCTGATCGCTAAAACCAACCAGCAGTTCGAACTGATCGACAACGAATTCAACG AAGTTGAAAAACAGATCGGTAACGTTATCAACTGGACCCGTGACTCTATCACCGAAGTTTGGTCTTACAACGCTGAACTGCTGATCGCTATGGAAAACCA GCACACCATCGACCTGGCTGACTCTGAAATGGACAAACTGTACGAACGTGTTAAACGTCAGCTGCGTGAAAACGCTGAAGAAGACGGTACCGGTTGCTTC GAAATCTTCCACAAATGCGACGACAACTGCATGGCTTCTATCCGTAACAACACCTACGACCACCGTAAATACCGTGAAGAAGCTATGCAGAACCGTATCC  AGATCGACCCGGTTAAACTGTCTTCTGGTTACAAAGACGTTATCCTGTGGTTCTCTTTCGGT |


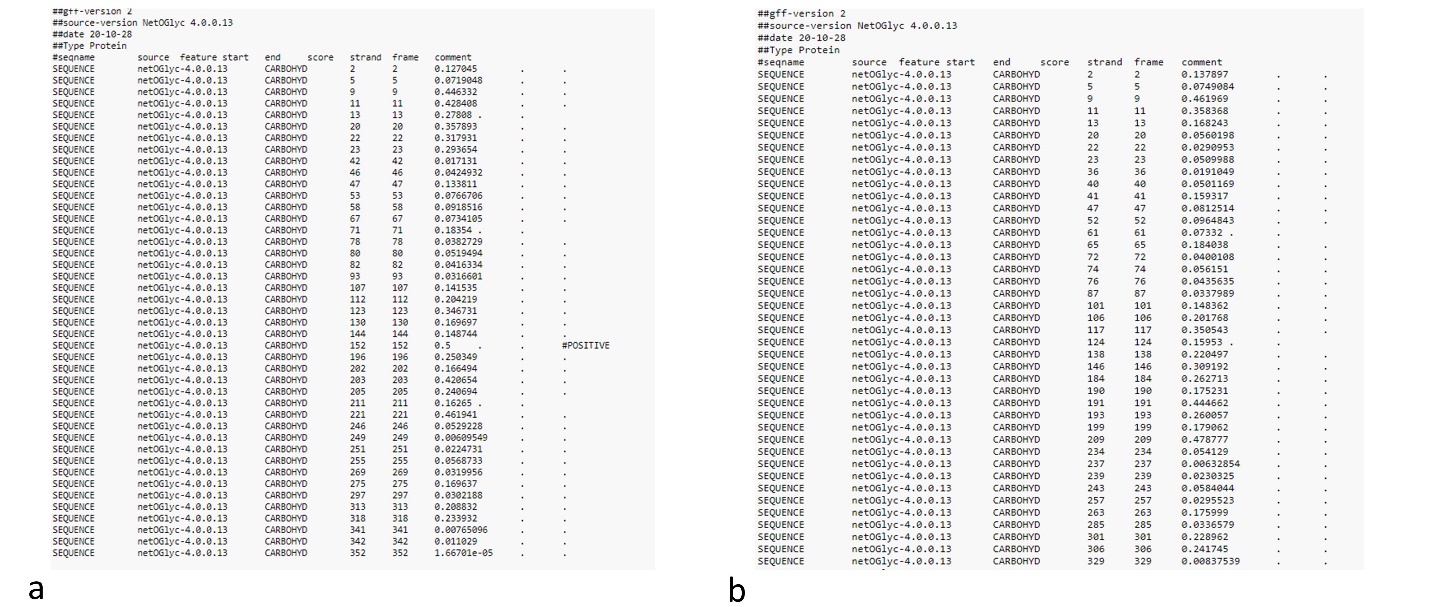


**Figure S1. O-glycosylation site prediction. Using NetOGlyC server, the potential O-glycosylation sites predicted for a) MCH-LR and b) MCH.**


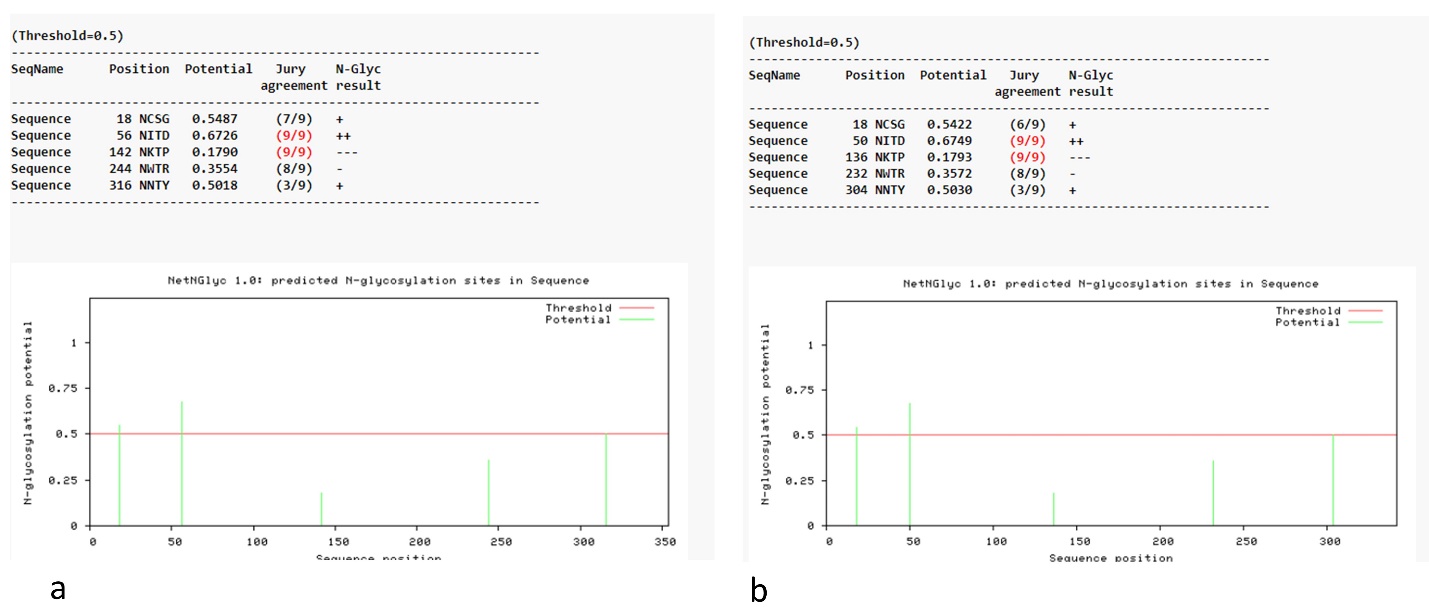


**Figure S2. N-glycosylation site prediction. Using NetNGlyC server, the potential N-glycosylation sites predicted for a) MCH-LR and b) MCH.**


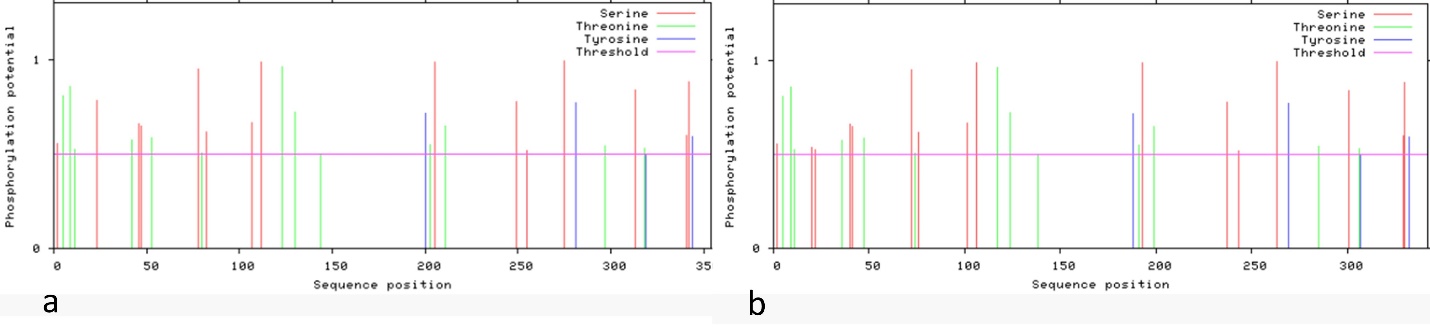


**Figure S3. Phosphorylation site prediction. Using NetPhos server, the potential phosphorylation sites predicted for a) MCH-LR and b) MCH.**

Table S14. Proteinases that potentially can cut the vaccine

| Thrombin | Pepsin (pH1.3) | Formic acid | Chymotrypsin-high specificity (C-term to [FYW], not before P) | Caspase4 | Arg-C proteinase |
| --- | --- | --- | --- | --- | --- |
| Trypsin | Pepsin (pH>2) | Glutamyl endopeptidase | Chymotrypsin-low specificity (C-term to [FYWML], not before P) | Caspase5 | Asp-N endopeptidase |
|  | Proline-endopeptidase [[*]](https://web.expasy.org/cgi-bin/peptide_cutter/peptidecutter.pl#pro_note) | Hydroxylamine | Clostripain | Caspase6 | Asp-N endopeptidase + N-terminal Glu |
|  | Proteinase K | Iodosobenzoic acid | CNBr | Caspase7 | BNPS-Skatole |
|  | Staphylococcal peptidase I | LysC | Enterokinase | Caspase8 | Caspase1 |
|  | Tobacco etch virus protease | LysN | GranzymeB | Caspase9 | Caspase2 |
|  | Thermolysin | NTCB (2-nitro-5-thiocyanobenzoic acid) | Factor Xa | Caspase10 | Caspase3 |
